# Dielectric Property and Space Charge Behavior of Polyimide/Silicon Nitride Nanocomposite Films

**DOI:** 10.3390/polym12020322

**Published:** 2020-02-04

**Authors:** Minghua Chen, Wenqi Zhou, Jiawei Zhang, Qingguo Chen

**Affiliations:** Key Laboratory of Engineering Dielectric and Applications (Ministry of Education), Harbin University of Science and Technology, Harbin 150080, China; zhouwenqi0827@163.com (W.Z.); jwzhang@hrbust.edu.cn (J.Z.)

**Keywords:** polyimide, silicon nitride, dielectric properties, space charge

## Abstract

Polymeric materials have many applications in multiple industries. In this paper, silicon nitride nanoparticles (Si_3_N_4_) were incorporated into a polyimide (PI) matrix to obtain composite films via the in situ polymerization method. The Si_3_N_4_ nanoparticles were consistently scattered in the composites, and the thickness of PI/Si_3_N_4_ films was around 50 µm. The effects of nanoparticle content on the dielectric constant, loss tangent and breakdown strength were simultaneously studied. A 3 wt.% doped PI/Si_3_N_4_ film revealled excellent dielectric properties, a dielectric constant (ε) of 3.62, a dielectric loss tangent (tanδ) of 0.038, and a breakdown strength of 237.42 MV/m. The addition of Si_3_N_4_ formed an interface layer inside PI, resulting in a large amount of space charge polarization in the electric field. The space charge of materials from the microscopic point of view was analyzed. The results show that there are trapenergy levels in the composites, which can be used as a composite carrier center and transport channel, effectively improving the performance of a small amount of nanoparticles film.

## 1. Introduction

Polyimide (PI) has been broadly used in the fields of electronic packing, the automotive and chemical industries, aerospace and precision machinery, etc. [1,2,3] due to its low dielectric constants, outstanding mechanical properties, high resistance to various temperatures (400 °C), excellent solvent resistance and good biocompatibility [4]. Furthermore, recent downsizing of current and cost reduction demands a reduction in its thickness. This means that the polyimide film is required to show a good performance as an insulating material under a high electric field and to keep the dielectric constant in some level. 

In order to achieve this goal, there have been great efforts to use high dielectric constant fillers, such as linear, flaky carbon materials or metal materials. In the literature, a lot of composite systems have been investigated, including different kinds of fillers (SiC [5], ZnO [6], AG [7], MWCNTs [8], Ni [9], CNTs and graphene oxide [10]), indicating that dropping nanofillers can effectively upraise dielectric constant. However, carbon and metal materials have high electrical conductivity, which seriously affects the insulating properties of the polymer, resulting in a dilemma that the dielectric strength and dielectric constant can only be improved.

To maintain a distance from this shortcoming, researchers have proposed that the ceramic materials (Ba_0.5_Sr_0.5_TiO_3_ [11], BaTiO_3_ [12,13]) would be excellent candidates for inorganic fillers. Among such a significant number of materials, silicon nitride (Si_3_N_4_) has attracted significant consideration owing to its captivating properties, such as wide band gap, excellent insulation performance, high quality at high temperature, and fantastic thermal conductivity [14]. Diaham et al. argued that the epoxy/Si_3_N_4_ (1 vol.%) nanocomposites show an improvement of the dielectric strength, by about 1.16 times, compared to pure epoxy [15]. Xu et al. obtained the polypropylene/Si_3_N_4_ (2 wt.%) and indicated that breakdown strength was increased by about 1.1 times [16]. He et al. suggested that the polystyrene/Si_3_N_4_ (40 vol.%) can increase the dielectric constant from 2.4 to 3.5 [17]. Yan et al. proposed that the Si_3_N_4_/epoxidized silane/cyanate ester can enhance the dielectric constant from 2.93 to 3.03 in 10 MHz [18]. Zhou et al. detailed that the polyethylene/Si_3_N_4_ (20 wt.%) increased the dielectric constant by about two times [19]. Previous research seems to be able to meet the requirements, which have increased the dielectric strength and dielectric constant of organic polymers by the doping of silicon nitride. Most of these works focused on dielectric and thermal properties, with less attention to the breakdown mechanism. There is still a lack of effective models and deep explanations for the mechanism of breakdown strength [20].

In this paper, we report an in situ polymerization process to prepare PI/Si_3_N_4_ nanocomposite films. The Si_3_N_4_ nanoparticles were consistently scattered in the composites, and the thickness of the PI/Si_3_N_4_ films was around 50 µm. Their electrical conductivity space charge distribution and thermal stability were studied systematically. An innovative attempt was made to explain the mechanism of breakdown by space charge. The PI/Si_3_N_4_ film with 3 wt.% Si_3_N_4_ substance introduced an exceptional heat resistance index of 279 °C, an excellent dielectric performance (ε and tanδ respectively from 3.3 to 3.2 and 0.0032 to 0.0112 at frequency range of 10^2^ to 10^6^ Hz), and a dielectric strength of 237.42 MV/m.

## 2. Experimental

### 2.1. Materials

Both 4,4′-diaminodiphenylether (ODA) and 1,2,4,5-benzenetetracarboxylic anhydride (PMDA) were acquired from Sinopharm Chemical Reagent Co., Ltd., Shanghai, China. *N*,*N*-dimethylacetamide (DMAC) was gotten from Tianjin in Fuyu Fine Chemical Co., Ltd., Tianjin, China. Silicon Nitride (Si_3_N_4_) was acquired from Shanghai Macklin Biochemical Co., Ltd., Shanghai, China, with a density of 3.44 g/mL and a particle size between 10 and 30 nm.

### 2.2. Preparation

**Figure 1:**
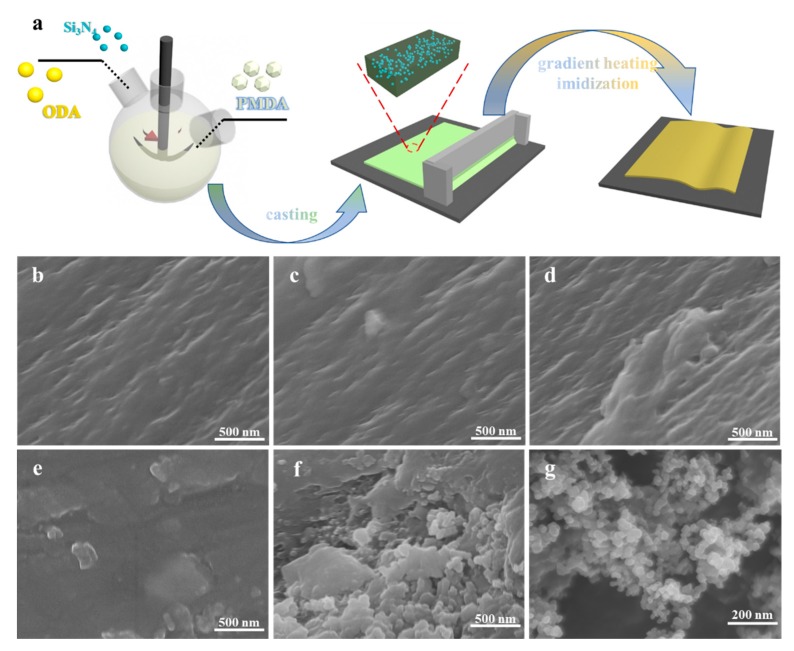
Preparation process of the PI/Si_3_N_4_ composites (**a**). SEM images of the cross-section of the pure polyimide (PI) (**b**), the 1 wt.% PI/Si_3_N_4_ (**c**), the 3 wt.% PI/Si_3_N_4_ (**d**), the 5 wt.% PI/Si_3_N_4_ (**e**), and the 7 wt.% PI/Si_3_N_4_ (**f**) nanocomposite films, and pure Si_3_N_4_ powder (**g**).

The films were prepared by a two-step method with various loadings of Si_3_N_4_. The schematic chart that addresses the readiness of PI/Si_3_N_4_ composites is displayed in Figure 1a. 

Firstly, we synthesized the poly(amide acid) (PAA) and PAA/Si_3_N_4_ precursors slurry. Initially, a certain amount of Si_3_N_4_ was scattered in 36 mL DMAC by sonication for a certain time. Along with DMAC, ODA (3 g, 0.013 mol) was added to a 250 mL three-necked flask outfitted with a mechanical stirrer. After the diamines were broken up, equimolar PMDA (3.28 g, 0.013 mol) was added in one portion to the flask. Consequently, the homogeneous viscous pulp of PAA (precursor of polyimide) and PAA/Si_3_N_4_ was obtained. Finally, this solution was cast on a glass substrate by a scraper blade. The second step was the thermal imidization process. To remove the residual solvent of DMAC, the film was placed in a vacuum oven at 80 °C for 4 h. After that, the composite was further thermally treated in a muffle furnace by the gradient heating method, from room temperature to 110, 140, 170, 200, 230, 260, 290, 320, and 350 °C for half an hour, then the PAA was changed over to PI totally. The PI and PI/Si_3_N_4_ nanocomposites (PI, PI/Si_3_N_4_-1, PI/Si_3_N_4_-3, PI/Si_3_N_4_-5 and PI/Si_3_N_4_-7) containing 0, 1, 3, 5 and 7 wt.% of Si_3_N_4_ were set up by the previously mentioned experimental processes.

### 2.3. Characterization

The microstructures of the composite films were taken on a SU8020 scanning electron microscope (SEM, Hitachi, Tokyo, Japan). Before testing, all the sample fracture surfaces were brittle broken by liquid nitrogen and coated with gold. Fourier transform infrared (FTIR) spectra were obtained on Perkin Elmer’s Spectrum Two L160000A, PerkinElmer, Waltham, MA, USA.

The dielectric constant (ε) and dielectric loss factor (tanδ) values of the samples (25 mm × 25 mm) were measured using Concept 80 at frequencies ranging from 10 to 10^6^ Hz. The samples were fabricated by sputtering a thin film aluminum pattern on both sides. The electric breakdown strength was measured using the HT-5/20A equipment with a 1 kV/s loading rate of voltage and the area of the copper electrode used was 3.14 cm^2^ at room temperature. The space charge distribution measurement of the specimen with an average thickness of 100 µm was performed in a pulse electro-acoustic (PEA) system. Silicone oil was used as acoustic coupling to ensure sound acoustic transmission between the specimen and the electrode. Calibration was conducted at a DC field of 3 kV/mm for 5 min to minimize the influence on space charge accumulation. Then, a DC electric field of 40 kV/mm was applied to the specimen for 30 min at room temperature and removed. The measurement signals, both in the polarization and depolarization process, were recorded over time, and the data were processed using a calibration trace and a deconvolution technique to restore the original signal. The thermogravimetric analysis (TGA) measurements were carried out using Pyris6 from 50 to 800 °C in the air at a heating rate of 10 °C/min. 

## 3. Results and Discussion

### 3.1. Structure Characterization of PI/Si_3_N_4_

Figure 1b–f separately shows the fracture morphologies of PI/Si_3_N_4_ composites with different Si_3_N_4_ mass contents from 1 to 7 wt.%. When the doping amount is meager, the dopants are evenly distributed in the polymer, and they are far away from each other. With the increasing of the dopant, the distance between the nanoparticles became closer and closer. Thus, the fracture morphologies of the PI/Si_3_N_4_ composites were much rougher. 

When the filler aggregated to a certain extent, the gap was almost gone, as depicted in Figure 1f. In addition, the disappearance of the gap leads to the formation of a conductive path and damages the insulation performance. This poor dispersion in high filler content was mainly attributed to the van der Waals force and the inevitable interfacial thermal barriers between fillers and the PI matrix. 

**Figure 2:**
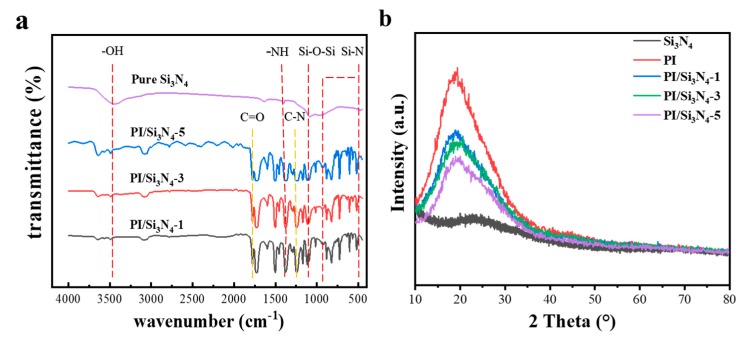
FTIR (**a**) and XRD (**b**) spectra of pure Si_3_N_4_ nanoparticles and PI/Si_3_N_4_ matrix.

The FTIR spectra of pure Si_3_N_4_ nanoparticles and PI/Si_3_N_4_ matrix are presented in Figure 2a. The absorption bands near 1650 cm^−1^ (amic acid C=O stretching) and 1550 cm^−1^ (amide C–N stretching) in PI/Si_3_N_4_ matrix are both disappeared, which proves that the thermal imidization of PAA is completed [21]. The bands near 1778 cm^−1^ and 1726 cm^−1^ can be ascribed to the asymmetrical and symmetrical stretching vibration of the C=O groups, respectively [22]. The characteristic stretching vibration peak at 1379 and 1116 cm^−1^ can be assigned to the C–N group, and the bending vibration peak at 720 cm^−1^ can be attributed to the imide group [23]. In addition, all the FTIR curves of the PI/Si_3_N_4_ complexes present clear characteristic absorption peaks near 3533, 1647, 1074, 952 and 447 cm^−1^, owing to the incorporation of Si_3_N_4_ nanoparticles [24,25].

As can be seen in Figure 2b, similar curves were observed for all the samples. It can be clearly seen that there is no special peak in silicon nitride, indicating that it is amorphous [16]. The diffraction peak at about 20° of PI decreased progressively with the addition of nano-silicon nitride, which demonstrated that nanoparticles had been successfully combined.

### 3.2. Thermal Properties of PI/Si_3_N_4_

**Figure 3:**
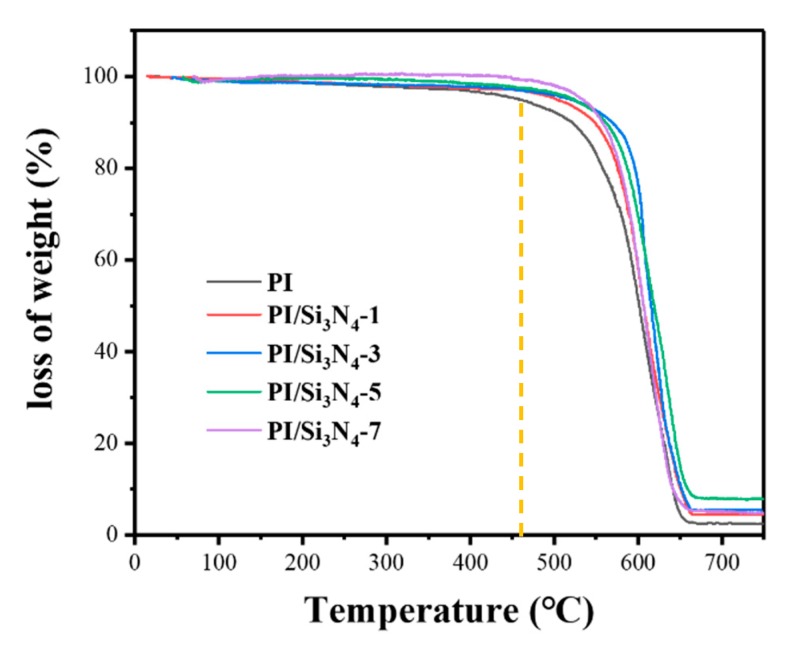
TGA analysis of PI and PI/Si_3_N_4_ films.

As can be seen from Figure 3 and Table 1, when the temperature is above 550 °C, the polymer begins to degrade rapidly, and the maximum degradation rate is around 600 °C. At the same time, the corresponding heat resistance index of pure PI and PI/Si_3_N_4_ composites are 259 °C, 272 °C (1 wt.%), 279 °C (3 wt.%), 279 °C (5 wt.%), 279 °C (7 wt.%), respectively. The results show that the thermal stability of the composites is improved, which is due to the high thermal conductivity of silicon nitride. 

### 3.3. Electrical Properties of PI/Si_3_N_4_

**Figure 4:**
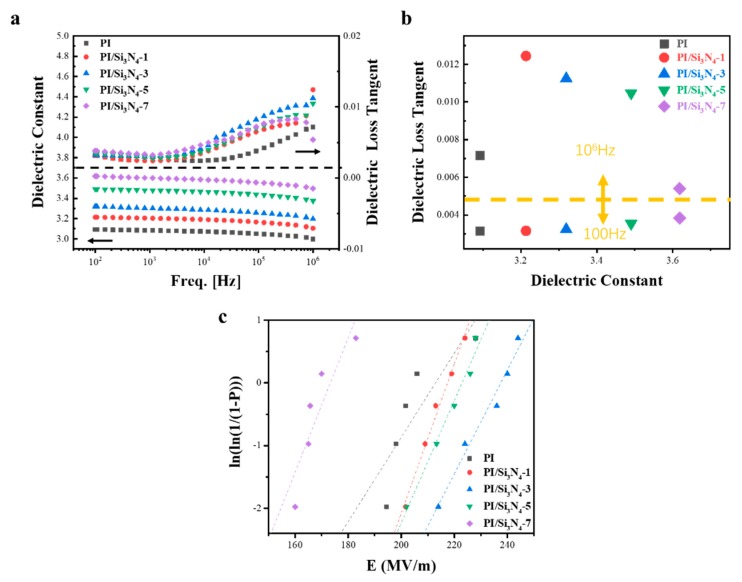
The mass fraction of Si_3_N_4_ fillers affecting (**a**) the dielectric constant (ε) and dielectric loss tangent (tanδ) values at a different frequency; (**b**) between dielectric constant (ε) and dielectric loss tangent (tanδ) values at 100 and 10^6^ Hz of the PI/Si_3_N_4_ composites; and (**c**) Weibull-distribution plot of breakdown strength for PI/Si_3_N_4_ composites.

Figure 4a demonstrates that both the dielectric constant (ε) and dielectric loss tangent (tanδ) were upgraded after dropping. The PI/Si_3_N_4_-7wt.% hybrid film exhibits a maximum dielectric constant of 3.62 at 100 Hz. More surprisingly, the dielectric loss tangent remains at a low level (tanδ = 0.0038). The quantity of orientation polarization groups for the PI/Si_3_N_4_ composites were increased step by step with the expanding mass fraction of Si_3_N_4_ fillers, beneficial to the improvement of the ε value. Furthermore, the dielectric loss tangent increased at the range of 10^5^ to 10^6^ Hz, which can be credited to the dielectric relaxation behaviors of polyimide. Dielectric loss alluded to the phenomenon that dielectric composites converted electrical energy into heat energy when subjected to an alternating electric field. It is worth noting that the dielectric loss of the complex shows almost no difference in low frequencies, and the value of PI/Si_3_N_4_-3 wt.% is especially outstanding. According to the micro capacitance theory [26], this phenomenon is mainly because the micro capacitance increases when the dopant content increases, leading to more significant dielectric loss. Due to the action of the external electric field, the dipole molecules in the medium repeatedly arrange and rub against each other as the frequency increases, resulting in polarization loss. The higher the dipole moment is, the higher the dielectric loss is.

The dielectric constant and dielectric strength of dielectric materials jointly determine their maximum stored energy, and the relationship of these three factors is shown in the following equation:

$$U_{e}=\frac{1}{2}\varepsilon_{0}{\varepsilon E}_{b}{}^{2}$$

where U_e_ is the discharged energy density, ε_0_ is the vacuum dielectric constant (ε_0_ = 8.85 × 10^−12^ F m^−1^), ε is the dielectric constant of the composite, and E_b_ is the breakdown strength of the complex. Since E_b_ is a quadratic independent variable, it has a greater impact on energy storage. Therefore, it is an effective way to improve the energy storage performance of materials to keep the dielectric constant constant or even increase while increasing the dielectric strength. We analyzed the strength of pure PI and composite films by using the two-parameter Weibull distribution function:

$$P=1-\exp\left[-{\left(\frac{E_{b}}{\alpha }\right)}^{\beta }\right]$$

where P is the cumulative probability of electric failure, E_b_ is the measured breakdown field, scale parameter α is the breakdown strength, for which there is a 63.2% probability for the sample to breakdown (Weibull E_0_), and shape parameter β is associated with the scattering of the data distribution. Let us take the logarithm of both sides of this equation:

$$\ln\left[-\ln\left(1-P\right)\right]=\beta \left({\mathrm{lnE}}_{b}-\ln\alpha \right)$$

In addition, for each measured value, the P is determined according to the following formula:

$$P=\frac{i-0.44}{n+0.25}$$

where i is the descending rank of the E value in the total breakdown sample data, and n is the total number of sample spaces [27].

In Table 2, all the PI/Si_3_N_4_ films have high β values, indicating that these composites were of a high quality and dependability. The breakdown strengths of these composites with various concentrations are plotted in Figure. 4c. It is obvious that the breakdown strength of PI/Si_3_N_4_-3 wt.% is 237.42 MV/m, 1.12 times higher than pure PI (212.42 MV/m). This is mainly because the ceramic filler itself exhibits high insulation, and the clustering phenomenon was not evident when the doping degree was lower than 7 wt.% (173.05 MV/m). The polyimide matrix with uneven packing distribution cannot evenly wrap Si_3_N_4_, resulting in defects and voids in the film, causing a distorted electric field distribution. As a result, the PI/Si_3_N_4_-7 wt.% composites will be divided into multilayers, which are conducive to develop breakdown strengths under the electric field. We can also see the correlation coefficients R in Table 2, which are all closer to 1, meaning that the analysis data for breakdown has a suitable fitting [28].

According to the intrinsic theory [29], the electric field will cause the breakdown of a material in a very short time, without the effects of high field aging. Under the action of the external electric field, there may be some electrons in the conduction band of the solid, and these electrons will gain energy movement due to the action of the external electric field, and the movement will interact with the lattice wave (phonons). When the energy gained by the electrons is much higher than the energy required to interact with lattice waves, their interaction will ionize new electrons, free electrons will increase, and then the breakdown phenomenon occurs.

**Figure 5:**
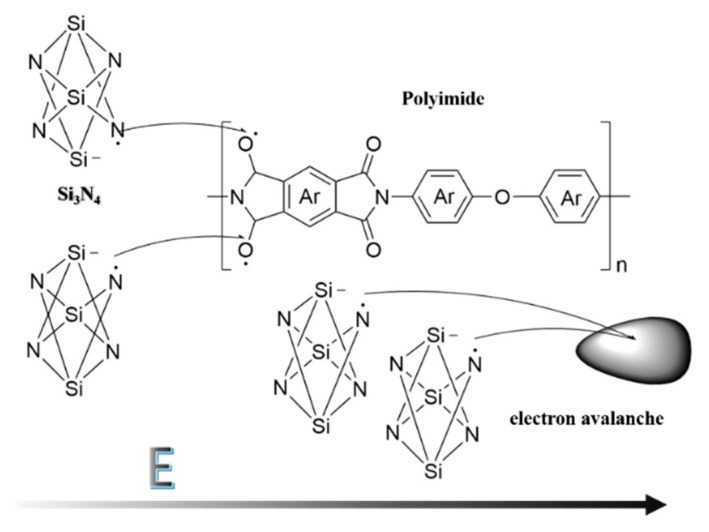
The model of electron avalanche for PI/Si_3_N_4_.

Due to the influence of the end group and impurity, the molecular structure will not be exactly ideal, and the electrons will not to be appear in pairs. As illustrated in Figure 5, when the external electric field acts, electrons that do not form covalent bonds will gain energy and become free electrons, which will move in the polymer along with the direction of the electric field. The free electrons have a certain probability of being captured by various elements, like Si, bringing about the breaking of the covalent bond and the appearance of extra electrons at the other end of the element, as N. What is more, on the grounds that the electric field is offering energy to these extra electrons, these atoms are going to snatch electrons from other atoms, such as O, shaping new covalent bonds. As the external electric field is strong enough, the energy of the excess electrons becomes larger and larger, and instead of forming covalent bonds, they bombard the molecular chain, causing ionization to produce secondary electrons and leading to electron collapse. Similarly, at the same field strength, when the dopant reaches a certain level, the polymer cannot adequately wrap the dopant, and will generate more excess electrons, which is more likely to cause electron collapse [30].

**Figure 6:**
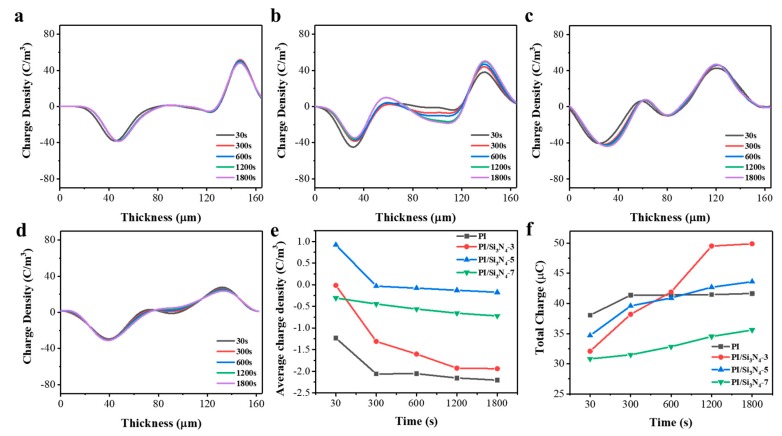
Space charge distribution in the pure PI (**a**), the 3 wt.% PI/Si_3_N_4_ (**b**), the 5 wt.% PI/Si_3_N_4_ (**c**) and the 7 wt.% PI/Si_3_N_4_ (**d**) nanocomposite films, and the curves of the average charge density of these (**e**) and the total charge quantity of these (**f**).

From the above inference, it is well known that the primary driver of a breakdown caused by electron collapse in the medium is the amassing of charge. Therefore, in order to understand the space charge characteristics of the complex, we selected the pure PI, the 3 wt.% PI/Si_3_N_4_, the 5 wt.% PI/Si_3_N_4_, and the 7 wt.% PI/Si_3_N_4_ nanocomposite films for space charge measurement (Figure 6). If the positive and negative charges in each repeating element in a homogeneous dielectric cannot counteract each other, the excess charge is the space charge at that position. In this paper, the space charge mainly refers to the trapped charge and the polarization charge caused by non-uniform polarization. 

As can be seen from Figure 6a, for pure polyimide films, the space charge is mainly distributed on the surface near the electrode, and the internal charge accumulation is almost zero. However, it is evident that a hetero-charge appears in the vicinity of electrodes (Figure 6b–d) in the composites. 

It can be seen from Figure 6e, with the time of applied electrical field increasing, the negative charge is obviously accumulated. However, the doping of Si_3_N_4_ reduces the negative space charge generated by the medium when the electric field is first applied, providing a positive space charge. At 3 wt.% doping, the positive and negative charges almost completely neutralize each other when the electric field is applied for 30 s. With the increase in applied time, the trend is basically the same as that of the undoped film. When the doped content is 5 wt.%, the positive charge accumulates for the most part at 30 s of the electrical field being applied. With the applied time increase, the positive and negative charges almost completely cancel out, thus the change is not obvious, and the average positive and negative charge density remains around 0. When the doped content is 7 wt.%, the change in the average charge density does not show a linear function relationship, and the change range is the least.

As can be seen from Figure 6f, the total charge of the undoped pure film is the most when the electric field is applied for 30 s, and the total charge does not change after the electric field is applied for 300 s. The 3 wt.% doped film rapidly accumulates charge before applying the electric field for 1200 s. Combined with Figure 6e, it can be seen that the negative charges are rapidly accumulating, and no charge accumulates from 1200 s to 1800 s. The 5 wt.% doped film accumulates space charge from 30 s to 1800 s, only the accumulation rate is slower than 3 wt.% doping. The 7 wt.% doped and 5 wt.% doped film accumulation process is similar, but the speed is slower. This phenomenon can be attributed to the trap effect of the medium.

**Figure 7:**
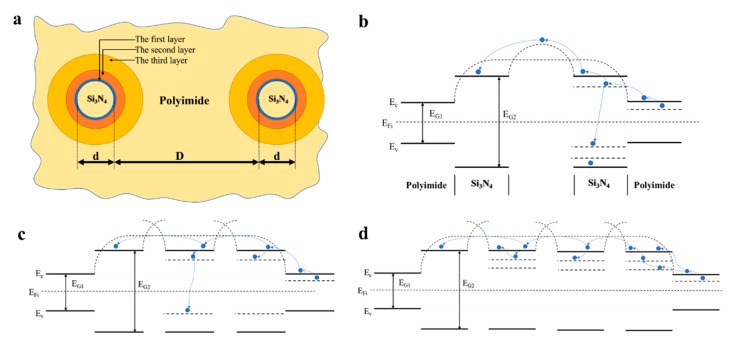
Schematic model of multi-core (**a**) and energy band structure for PI/Si_3_N_4_ composite films in different contents: 3 wt.% (**b**), 5 wt.% (**c**), 7 wt.% (**d**).

As Tanaka supposed [31], a multi-core model of the interface of a spherical inorganic filler nanoparticles doped in polyimide is shown in Figure 7a. The first layer is a bonded layer, which consists of combined filler and polyimide matrix by ionic, covalent or hydrogen bonds. The second layer is a bound layer dependent on the van der Waals force and is the main layer of polymer conformation. These two layers present deep traps. The third layer is considered to be a region affected chemically by the second layer, and electrically by the diffuse Gouy–Chapman layer to form a somewhat amorphous morphology, and is the main source of shallow traps. According to band theory [32], there is a forbidden band (E_G1/2_) between the conduction band (E_c_) and valence band (E_v_) of the semiconductor and insulator. Trap levels appear between the conduction band and valence band which are mainly derived from end groups, molecular chain breaks, branched chains, dielectric impurities, interfaces of different chemical clusters, amorphous interfaces of crystals and intermolecular chains of polymers etc.

According to the literature [33], the bandgap of polyimide is around 2.9 eV and will decrease with the increasing of chain length. Meanwhile, the bandgap of Si_3_N_4_ is around 6.9 eV [34]. As shown in Figure 7b–d, there is a wide and high barrier between adjacent cells in pure polyimide. The potential barrier is higher when the silicon nitride nanoparticles contact the polyimide, due to its wide band gap and small doping amount. Thus, charge carriers do not have enough energy to pass through the barrier, which makes it easy to be caught by deep traps and difficult to escape. If the doping ratio increases, the spacing between the particles will become smaller. The second and third layer is larger than the first layer, and the density of shallow traps is larger. Thus, the retention time of trapped charges is smaller, and the potential barrier is lower for mobile charge carriers. The energy obtained by charge carriers from the applied field would be increased. The polymer chain fracture may become easy, causing a lower breakdown field.

## 4. Conclusions

In summary, polyimide composites containing Si_3_N_4_ nanoparticles were successfully obtained by the in situ polymerization method. A 3 wt.% doped PI/ Si_3_N_4_ film revealed an excellent dielectric performance (ε and tanδ respectively from 3.3 to 3.2 and 0.0032 to 0.0112 at frequency range of 100 to 10^6^ Hz, and dielectric strength 237.42 MV/m). The space charge measurement results show that the accumulated charge density is almost negligible at the initial voltage in the PI/Si_3_N_4_-3% film. With the increasing of the doping amount, the initial accumulation of positive charge increases, completely suppressing the negative charge accumulation of the PI chain. The relationship between the breakdown strength and the space charge is based on the electronic transport in the nanoscale system, considering the large surface area of nanoparticles and the novel characteristics of the interface regions.

## Figures and Tables

**Table 1:** Characteristic thermal data of PI and PI/Si_3_N_4_ composites.

Samples	Weight Loss Temperature (°C)		*T*_heat-resistance index_^1^ (°C)	*T*_max_ (°C)
	5%	30%		
PI	454.6	577.8	258.9748	607.3
PI/Si_3_N_4_-1	502.5	589.5	271.803	611.6
PI/Si_3_N_4_-3	519.4	603.2	279.1432	613.4
PI/Si_3_N_4_-5	524.4	599.3	278.9766	637.1
PI/Si_3_N_4_-7	538.8	590.2	279.1236	625.4

^1^*T*_heat-resistance index_ = 0.49 × [*T*_5_ + 0.6 × (*T*_30_ − *T*_5_)], *T*_5_, *T*_30_ is the decomposing temperature at 5%, 30% weight loss, respectively.

**Table 2:** Linear fitting results and Weibull parameters of PI composite films.

Samples	Linear Fitting Results			Weibull Parameters	
	Slope	ln[−ln(1−P)] Intercept	R	β	E_0_
PI	14.392	−77.122	0.75	14.392	212.42
PI/Si_3_N_4_-1	25.346	−136.4	0.99	25.346	217.36
PI/Si_3_N_4_-3	18.91	−103.43	0.97	18.91	237.42
PI/Si_3_N_4_-5	20.871	−112.84	0.98	20.871	222.84
PI/Si_3_N_4_-7	18.601	−95.86	0.82	18.601	173.05
